# Microbiotherapy: an emerging adjunct for burn wound healing

**DOI:** 10.3389/fcimb.2026.1873498

**Published:** 2026-07-20

**Authors:** Junxiao Zhang, Kangrui Zhang, Yiran Wang, Guojuan Fan, Yanna Lv, Jinlong Ma

**Affiliations:** 1 School of Pharmacy, Shandong Second Medical University, Weifang, Shandong, China; 2 Dermatology, Weifang Hospital of Traditional Chinese Medicine, Shandong Second Medical University, Weifang, Shandong, China; 3 Shandong Engineering Research Center for Smart Materials and Regenerative Medicine, Shandong Second Medical University, Weifang, Shandong, China

**Keywords:** bacteriophage, burn, burn wound healing, microbiotherapy, probiotics

## Abstract

Burn injuries rank among the most common types of severe trauma, typically causing damage to skin tissue. However, traditional management strategies, particularly those relying on broad-spectrum antibiotics, face the dual challenges of increased drug resistance and limited wound healing efficacy, necessitating urgent development of novel therapeutic strategies. In recent years, microbiotherapy has garnered significant attention as a potential adjuvant therapy in burn wound management. Microbiotherapy achieves antibacterial effects by competitively inhibiting pathogen adhesion and secreting metabolites. Additionally, it promotes burn wound healing through the secretion of anti-inflammatory and growth factors, as well as the restoration of immune homeostasis. This approach aligns with the field’s shift toward personalized therapy, where microbial interventions can be tailored to the specific microenvironment of a patient’s wound. Current microbiotherapies for burn treatment primarily focus on probiotics, bacteriophages, and microbial metabolic derivatives. These therapies can be used individually or in combination and are gradually transitioning from laboratory early-stage research to clinical application. Probiotics, the core component of microbiotherapy, exert their therapeutic effects mainly by secreting various metabolites. This review summarizes the latest advances in microbiotherapy for burn wound treatment, focusing on its mechanisms of action, *in vivo* studies, and potential combined applications. Furthermore, this review critically evaluates the future opportunities and translational challenges of microbiotherapy to provide a balanced perspective on its clinical viability.

## Graphical Abstract

**Figure:**
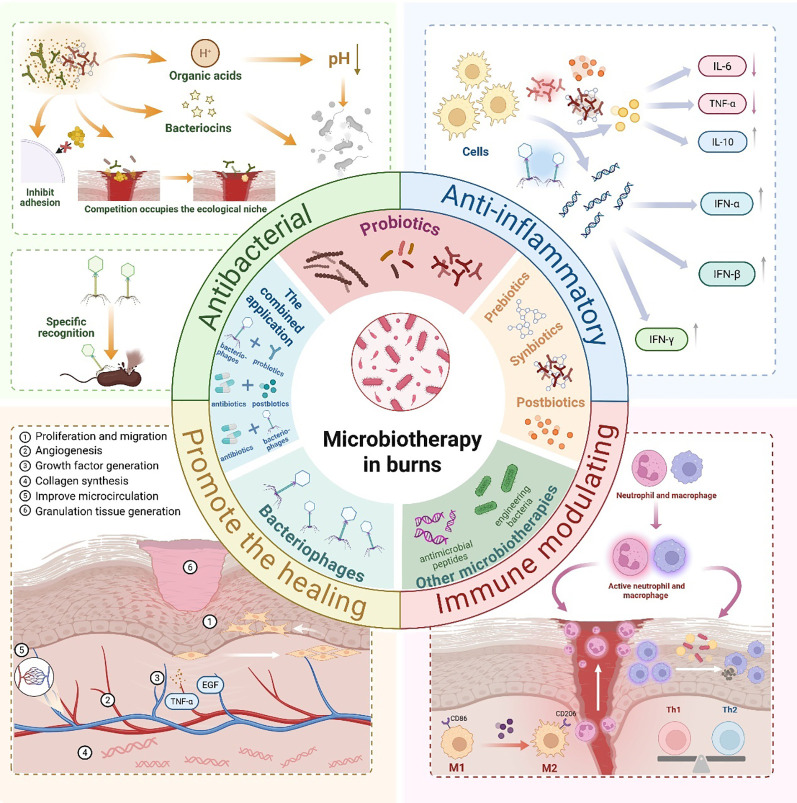
Schematic diagram illustrating the mechanism by which microbiotherapy treats burns. By Biorender.com.

## 1 Introduction

The human skin serves as a home for various microorganisms like bacteria, fungi, and viruses, forming a complex ecosystem known as skin microbiota. Skin microbiota have dual roles—one as a physical barrier that protects the skin from pathogens, and the other as a regulator that maintains skin health via immune modulation (Harris-Tryon and Grice, 2022). However, the occurrence of burns severely impairs this protective barrier. Burns are serious injuries to the skin or other tissues caused by heat, chemicals, electricity, or other external factors, and usually lead to pain, swelling, skin damage, and even tissue necrosis (Żwierełło et al., 2023). Burns not only inflict physical harm but also result in permanent scars after healing, which can have a significant impact on an individual’s self-esteem and social interactions (Jeschke et al., 2020). According to the World Health Organization (WHO)’s 2023 data, around 11 million people worldwide suffer burns each year, and approximately 180,000 of these cases result in death. Therefore, strengthening burn treatment is critical for reducing casualties, improving patients’ quality of life, and easing the social burden.

In burn wound treatment, the formation of biofilms is one of the major reasons for treatment failure (Sauer et al., 2022). The current mainstream approach to burn treatment involves wound dressings and surgical grafting combined with antibiotic therapy. However, traditional antibiotic drugs cannot easily penetrate the biofilm to reach internal bacteria, and are likely to trigger drug resistance problems (Hemmati et al., 2023). The presence of biofilms not only increases bacterial resistance to traditional antibiotics and host immune responses nearly 1,000 times, but also causes more than 80% of bacterial infections to be difficult to clear (Vishwakarma et al., 2021). Consequently, the field is urgently moving beyond broad-spectrum antibiotics toward better ways to manage wound infection that are both targeted and adaptive. Additionally, surgical therapy procedures frequently encounter problems, including a lack of donor skin, an increased risk of infection, and a slower recovery time after the procedure (Schlottmann and Lorbeer, 2024). In fact, burn treatment requires more than just simple antibacterial therapy and skin grafting. It is a complex, systematic recovery process encompassing multiple stages, including antibacterial, anti-inflammatory, wound healing promotion, and immune modulating (Scheme 1). To overcome these limitations and address the multifaceted demands of burn wound healing, microbiotherapy has emerged as a promising alternative.

Microbiotherapy is a medical approach that uses organisms or their derivatives to prevent or treat diseases. Compared to traditional methods, it has advantages like high specificity, low toxicity, and the ability to promote tissue repair (Kogan et al., 2019). As burn care transitions toward personalized therapy approaches, microbiotherapy provides a unique platform to modulate the wound environment based on individual microbial profiles. Among multiple microbial agents, probiotics dominate the research of burn wound microbiotherapy, and their biological functions are largely dependent on diverse metabolites secreted during growth and metabolism (Ambrose et al., 2025). During the burn wound healing process, microbiotherapy such as probiotics and bacteriophages is being increasingly investigated as a promising adjuvant strategy. These targeted agents have demonstrated excellent ability to identify and eliminate pathogens in experimental studies. They can also play a positive role in promoting tissue repair and reducing the risk of infection by regulating the expression of inflammatory factors, growth factors, and immune cells (Piranaghl et al., 2023; Molendijk et al., 2024; Wang et al., 2026). By integrating these microbial strategies as adjuvants to standard care, clinicians are expected to achieve more precise infection control and accelerated tissue repair. These capabilities provide patients with safer and more precise treatment options, and also open up new avenues for the development of burn treatment.

## 2 The mechanism of microbiotherapy

Microbiotherapy, by targeting the intricate burn wound microenvironment, offers a novel perspective for burn management. The advantages behind it are not solely the result of a single effect, but stem from its multidimensional mechanism of action.

### 2.1 Probiotic metabolites: major classes and core mechanisms

Probiotic metabolites are the key functional substances that mediate the therapeutic effects of probiotics on burn wounds. After colonizing the wound surface, probiotics continuously synthesize and secrete a variety of active metabolites into the microenvironment. These substances act as direct effectors to execute antibacterial, anti-inflammatory, immune-regulatory and pro-healing functions, and are the material basis for probiotics to participate in the whole process of burn wound repair (Hajialibabaei et al., 2025; Wilson et al., 2025). The table below summarizes the main categories of these metabolites and their primary therapeutic functions (**Table 1**).

**Table 1:** Major classes of probiotic metabolites and their therapeutic functions.

Category	Representative substances	Core therapeutic mechanism
Organic acids Small molecular compounds Bacteriocins Proteins/EVs Small RNA	Lactic acid, acetic acid, etc. SCFAs, H_2_O_2_, EPS, etc. Nisin A, etc. *Lactobacillus* EVs, NOD2 ligand proteins, etc. sRNA71, etc.	Modulate local pH, inhibit pathogen growth, regulate macrophage polarization and inflammatory responses Immune homeostasis, barrier reinforcement, antioxidant defense Target and destroy pathogen cell membranes, inhibit cell wall synthesis Deliver bioactive substances, promote cell activation, angiogenesis, collagen deposition, regulate immunity and tissue regeneration Modulate host gene expression and signaling pathways, regulate cell proliferation, migration and signal transduction

#### 2.1.1 Organic acids

Organic acids, important metabolic mediators derived from probiotic bacteria, link bacterial secretions to host signaling. These metabolites exert immunomodulatory effects through multiple pathways, including extracellular pH modulation and direct regulation of macrophage inflammatory phenotypes (Zhou et al., 2022). Probiotics generate these organic acids as major fermentation metabolites, establishing a direct metabolic connection between microbial colonization and host immune regulation (Tejero-Sariñena et al., 2012). A core environmental effect of organic acids is extracellular acidification, which creates a selective growth advantage for beneficial bacteria while inhibiting pathogenic colonization through competitive exclusion—a phenomenon termed the “priority effect” in microbial ecology (Yang et al., 2025a). Meanwhile, lactic acid and related monocarboxylic acids are transported into host immune cells via proton-coupled monocarboxylate transporters (MCTs). This co-transport of protons and lactic acid alters intracellular pH and modulates glycolytic flux, which contributes to macrophage polarization and inflammatory regulation (Choi et al., 2025). Specifically, lactic acid acts as a signaling molecule by binding to the G-protein-coupled receptor 81 (GPR81, also termed hydroxycarboxylic acid receptor 1, HCAR1), thereby activating AMP-activated protein kinase (AMPK) and its downstream effector large tumor suppressor kinase 1 (LATS1). This signaling cascade induces phosphorylation and inactivation of yes-associated protein (YAP), disrupting its interaction with nuclear factor kappa B (NF-κB) subunit p65 and attenuating NF-κB-driven excessive pro-inflammatory responses (Yang et al., 2020; Zhang et al., 2024). In parallel, acetic acid modulates this immunomodulatory network by activating G-protein-coupled receptor 43 (GPR43, also termed free fatty acid receptor 2, FFAR2) to curb Nucleotide-binding oligomerization domain-like receptor family pyrin domain containing 3 (NLRP3) inflammasome signaling. Notably, lactic acid represses NF-κB-mediated inflammatory responses through GPR81, while acetic acid engages GPR43 to suppress NLRP3 inflammasome in a calcium-dependent manner. The two receptors mediate complementary anti-inflammatory pathways to limit aberrant immune activation and maintain tissue immune homeostasis, alleviating inflammatory damage and promoting tissue repair (Xu et al., 2019).

#### 2.1.2 Small molecular compounds

Small molecular compounds, including short−chain fatty acids (SCFAs), hydrogen peroxide (H_2_O_2_), and exopolysaccharides (EPS), constitute a versatile group of probiotic−derived metabolites that integrate epigenetic regulation, antioxidant defense, and immune modulation to maintain tissue homeostasis (Demir and Aslim, 2025; Zhao et al., 2025). Short-chain fatty acids, with butyrate as a representative functional member, serve as histone deacetylase (HDAC) inhibitors. Butyrate functions in host epithelial cells to restrain HDAC enzymatic activity, thereby sustaining histone acetylation and an open chromatin conformation, which facilitates the transcription of genes responsible for maintaining barrier integrity (Schilderink et al., 2013). Probiotics can produce H_2_O_2_ during metabolism to participate in epithelial repair. As a typical probiotic genus, *Lactobacillus* generates low concentrations of H_2_O_2_, which promotes epithelial restitution (Singh et al., 2018). Multiple bioactive metabolites secreted by *Lactobacillus* strains can trigger the nuclear factor erythroid 2-related factor 2 (Nrf2) signaling cascade in cellular models, which upregulates antioxidant proteins and enzymes including heme oxygenase-1 (HO-1), NAD(P)H quinone dehydrogenase 1 (NQO1), superoxide dismutase (SOD) and catalase (CAT). These antioxidant effectors scavenge excessive reactive oxygen species (ROS), alleviate cellular oxidative stress and mitigate oxidative tissue damage, which contributes to optimizing the local microenvironment and facilitating tissue recovery (Li et al., 2022b). Moreover, probiotic exopolysaccharides (EPS) perform immunomodulatory and anti-infective activities through pattern recognition receptor interactions. *Bacillus subtilis* EPS engages Toll-like receptor 4 (TLR4) on immune cells to induce the inhibitory molecule IDO in dendritic cells, thereby activating the kynurenine/aryl hydrocarbon receptor (AhR) circuit that suppresses T cell proliferation and promotes anti-inflammatory macrophage polarization (Zamora-Pineda et al., 2023).

#### 2.1.3 Bacteriocins

Bacteriocins exhibit targeted antibacterial activity relying on their unique structural characteristics. Nisin A is one of the most extensively studied representative bacteriocins and acts through a typical docking mechanism against susceptible bacteria. This bacteriocin specifically recognizes and binds the pyrophosphate group of bacterial Lipid II, a vital peptidoglycan precursor required for bacterial cell wall synthesis (Breukink et al., 1999; Dickman et al., 2019). Stable non-covalent binding between Nisin A and Lipid II exerts dual bactericidal effects: it directly inhibits cell wall biosynthesis, while also promotes the insertion of hydrophobic structural segments into bacterial lipid bilayers. This process drives the assembly of transmembrane pore structures on bacterial membranes. The pore formation disrupts bacterial membrane potential and increases ion permeability, ultimately interfering with bacterial physiological metabolism and inducing bacterial death (Wiedemann et al., 2001). Notably, this antibacterial mode does not depend on conventional protein receptors, which enables bacteriocins to specifically target pathogenic bacteria with minimal impact on host cells (Cotter et al., 2005).

#### 2.1.4 Proteins/extracellular vesicles

Probiotic high-molecular-weight active metabolites mainly include functional proteins and extracellular vesicles (EVs). As nanoscale lipid bilayer vesicles, EVs encapsulate proteins, nucleic acids and small-molecule metabolites and deliver these cargos into recipient host cells via endocytosis (Yamasaki-Yashiki et al., 2023). Once internalized, the carried functional proteins and nucleic acids can mediate multiple host cellular biological processes, including the regulation of redox signaling, inflammatory responses, and cellular senescence (Amico et al., 2026). Multiple verified animal wound models have clarified the repair functions of probiotic-derived EVs, covering multiple classically recognized probiotic genera. EVs secreted by *Lactobacillus druckerii* activate keratinocytes and fibroblasts, boost angiogenesis and collagen deposition, suppress pathogenic bacteria proliferation, and accelerate tissue regeneration in murine full-thickness infected cutaneous wounds (Qi et al., 2024). For chronic wounds, hydrogel loaded with *Lactobacillus bulgaricus*-derived EVs exerts anti-inflammatory and pro-regenerative effects, which remodel disordered inflammatory microenvironment and stimulate granulation tissue formation to achieve efficient wound closure (Yuan et al., 2025). Beyond lactobacilli, the probiotic *Bacillus subtilis* also generates functional EVs. Its vesicles contain NOD2 ligand proteins that activate RIPK2 signaling to facilitate epithelial cell migration and wound repair *in vitro* (Baerg et al., 2024). The functional cargo inside probiotic EVs coordinately regulates the whole process of skin regeneration: it balances local cutaneous immune homeostasis, drives epidermal cell proliferation and capillary angiogenesis, and coordinates multiple signaling cascades to promote ordered skin tissue reconstruction.

#### 2.1.5 Small RNA

Probiotic bacteria secrete small RNAs (sRNAs) that may enter host cells through endocytic pathways, representing an emerging mechanism of interkingdom communication (Yamasaki-Yashiki et al., 2023). A notable example is sRNA71 from *Lactobacillus plantarum*, which was shown to reduce Tp53 expression in HEK293T cells through direct binding to the 3’ untranslated region of Tp53 mRNA, as demonstrated by luciferase reporter assay and cellular proteomics (Yu et al., 2022). This finding suggests that microbial sRNAs are capable of modulating host gene expression. Beyond direct gene targeting, sRNA species encapsulated within probiotic-derived EVs may modulate host cellular responses through alternative mechanisms, including regulation of transforming growth factor-β (TGF-β)/Smad and mitogen-activated protein kinase (MAPK) signaling pathways implicated in epidermal homeostasis and repair processes (Qin et al., 2024). These preliminary findings suggest that probiotic sRNAs may contribute to host cellular homeostasis, though their specific roles in cutaneous repair require further investigation.

### 2.2 Antibacterial effect

Burn wounds rich in proteins, plasma exudates, and necrotic tissues act as pathogen “media” (Church et al., 2006). Burn infections involve various pathogens, mainly *Staphylococcus aureus* (*S. aureus*) and *Pseudomonas aeruginosa* (*P. aeruginosa*). *S. aureus* worsens tissue damage and infection spread by secreting multiple toxins, with infected wounds showing redness and pus discharge. If untreated, these infections can spread through the bloodstream to cause sepsis (Brandenburg et al., 2021). *P. aeruginosa* often causes invasive necrotizing infections, with resistance linked to its outer membrane and low permeability, and it produces β-lactamase to resist carbapenem antibiotics. Additionally, *Acinetobacter baumannii* (*A. baumannii*) has emerged as one of the primary pathogens responsible for widespread transmission in intensive care units due to its multidrug resistance (MDR) and exceptional environmental adaptability (Jiang et al., 2022). Due to the high drug resistance and pathogenicity of these bacteria, patients’ hospital stay will be prolonged and the risk of worsening the condition will also increase. In this context, microbiotherapy has been investigated as a potential strategy to address these challenges through multidimensional antibacterial mechanisms.

The antibacterial effect is the most common and crucial mechanism of microbiotherapy, playing a vital role in burn wound treatment. Microbiotherapy combats pathogenic microorganisms such as bacteria and fungi by directly killing them or inhibiting their growth and reproduction (Chen et al., 2020). Probiotics can secrete multifunctional antibacterial substances such as organic acids and bacteriocins, which directly inhibit the growth and reproduction of pathogens. Indirectly regulating the microenvironment to achieve antibacterial effects is also an indispensable dimension in the antibacterial mechanism of microbiotherapy. Among them, prebiotics play a particularly typical role—they provide “nutritional support” for probiotics, promoting large-scale reproduction of probiotics on wound surfaces and occupying dominant colonization sites, thereby competing with pathogenic bacteria for survival space and preventing their attachment and spread from the source (Asahara et al., 2016). This indirect approach complements direct antibacterial methods, and together they build a dual antibacterial system for microbiotherapy.

**Figure 1:**
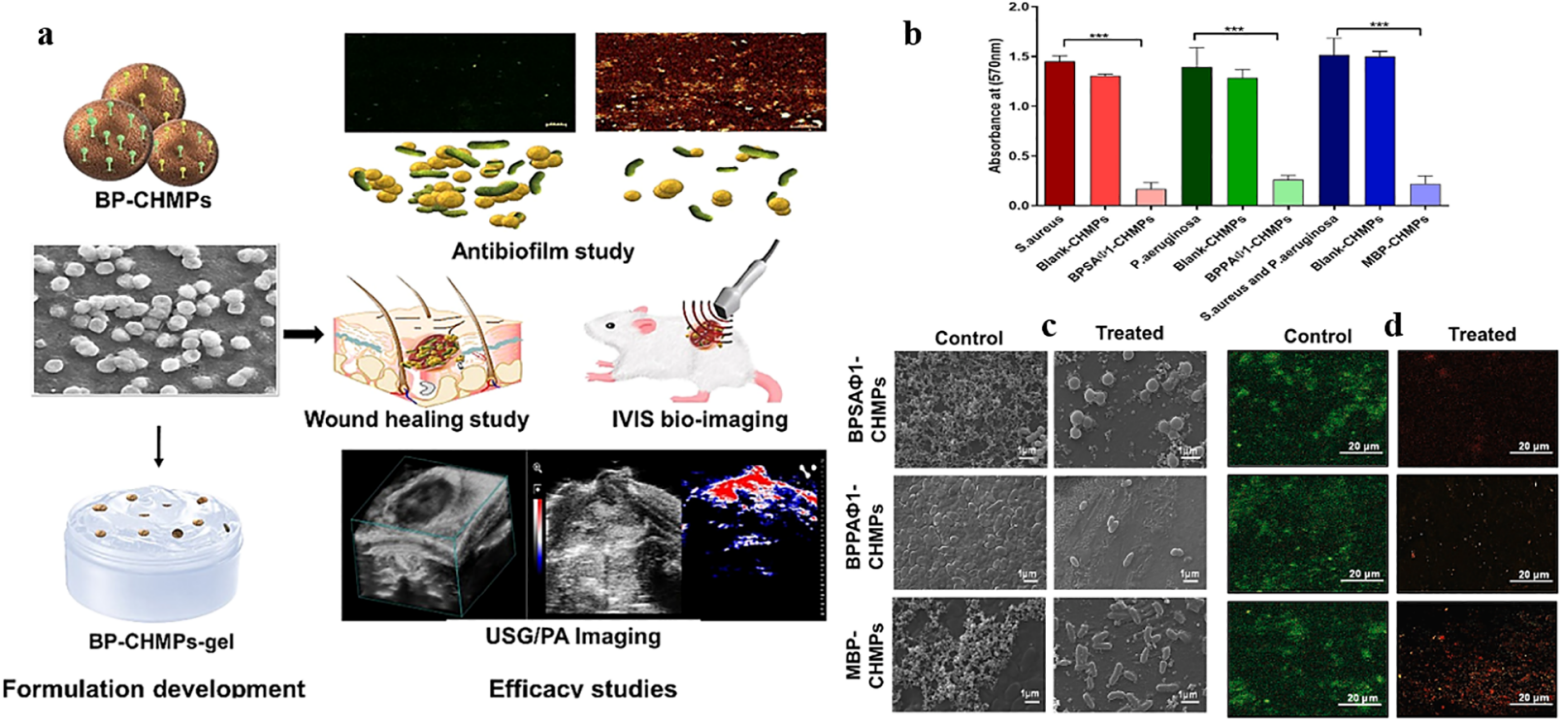
Antibiofilm effect of bacteriophage formulations on *S. aureus, P. aeruginosa* and a mixed bacterium (both). **(A)** Schematic diagram of formulation development and efficacy studies. **(B)** Treatment effect of different bacteriophage (BP) formulations on biofilms. **(C)** Scanning electron microscopy of untreated and treated biofilms. **(D)** Confocal laser scanning microscopy (green is indicative of live and red is indicative of dead bacteria). Reprinted from Ref Dehari et al. (2023b) with permission.

Bacteriophages, as special microbial agents, can specifically recognize and lyse the bacteria involved in infected wound sites (**Figure 1**), directly reducing pathogen load, thereby lowering lipopolysaccharide, peptidoglycan and other bacterial components’ continuous stimulatory effect on host immune systems (Dehari et al., 2023b). Compared to traditional antibiotics, bacteriophage therapy shows high specificity for targeting bacteria and thus avoids interfering with other human cells and beneficial microflora (Azevedo et al., 2022; Borzilov et al., 2025). Microbiotherapy can make use of the natural biological characteristics of these agents to comprehensively optimize the wound microenvironment and control bacterial infection from multiple aspects. Its antibacterial effects have demonstrated potential in managing burn wound infections and improving treatment outcomes in experimental models, potentially reducing the risk of complications. However, most of the current antibacterial studies are still limited to *in vitro* experiments or simplified rodent models, and there is a lack of complex infection models and human research data closer to clinical real scenarios (Yang et al., 2025b). Although *in vitro* experiments can effectively evaluate the antibacterial activity and preliminarily verify the mechanism of action, they cannot simulate the complex physiological microenvironment, immune response and microbial interaction *in vivo*. The conventional rodent model is mostly a short-term observation under a single strain infection, and it is difficult to restore the real situation of burn wound skin barrier damage and multi-strain mixed infection (Ojeh et al., 2025; Poghosyan et al., 2026). In addition, there are still key knowledge gaps about how microbial agents penetrate and destroy highly complex multi-microbial biofilms and their protective matrix barriers in human clinical burn wounds, and changes in protease activity and pH in the burn wound environment often significantly interfere with the survival rate and activity of microbial agents. These limitations greatly limit the effective transformation from basic research to clinical application.

### 2.3 Anti-inflammatory effect

Burn injuries often show a robust inflammatory response, serving as the body’s natural defense system against bacterial infections and cell injury from burns. In the early stage of burn injury, the body’s inflammatory response can prompt neutrophils, macrophages, and other immune cells to aggregate at the wound site. These cells can phagocytose invading pathogens, clear damaged tissue debris, and lay the foundation for subsequent wound healing (Mulder et al., 2022). However, excessive inflammatory responses trigger sustained release of pro-inflammatory cytokines, which impede keratinocyte migration and proliferation, delay granulation tissue formation, and exacerbate collagen degradation. This not only prolongs the wound healing process, but also causes further tissue damage, increasing the risk of scar hyperplasia and tissue necrosis (Shaw et al., 2023). As a therapeutic option for burns, microbiotherapy offers a core advantage in precisely regulating the expression and activity of inflammatory factors, thereby suppressing excessive inflammation.

Specific probiotics and their metabolites, for example, can lower the expression of key factors such as tumor necrosis factor-alpha (TNF-α) and interleukin-6 (IL-6), thereby reducing the inflammatory response (Han et al., 2024). Probiotics can also promote precise regulation of the inflammatory response by coordinating with the regulation of intestinal immunity and local defense mechanisms (Algieri et al., 2021). Bacteriophages show important anti-inflammatory effects in microbiotherapy, as they are able to regulate the immune response by inducing the release of interferons (Cao et al., 2022). For example, IFN-γ can enhance antibacterial immune response and moderately regulate the intensity of inflammatory response, thus maintaining balance during the wound healing process (García et al., 2019). This not only reduces the risk of wound infection, but also helps slow down tissue damage caused by excessive inflammation, contributing to a more favorable environment for wound healing. While these results underscore the anti-inflammatory potential of microbial agents, more critical research is needed to determine the optimal timing for applying these microbial agents, as premature suppression of inflammation might inadvertently hinder the initial debridement phase of wound healing.

### 2.4 Promotion of wound healing

**Figure 2:**
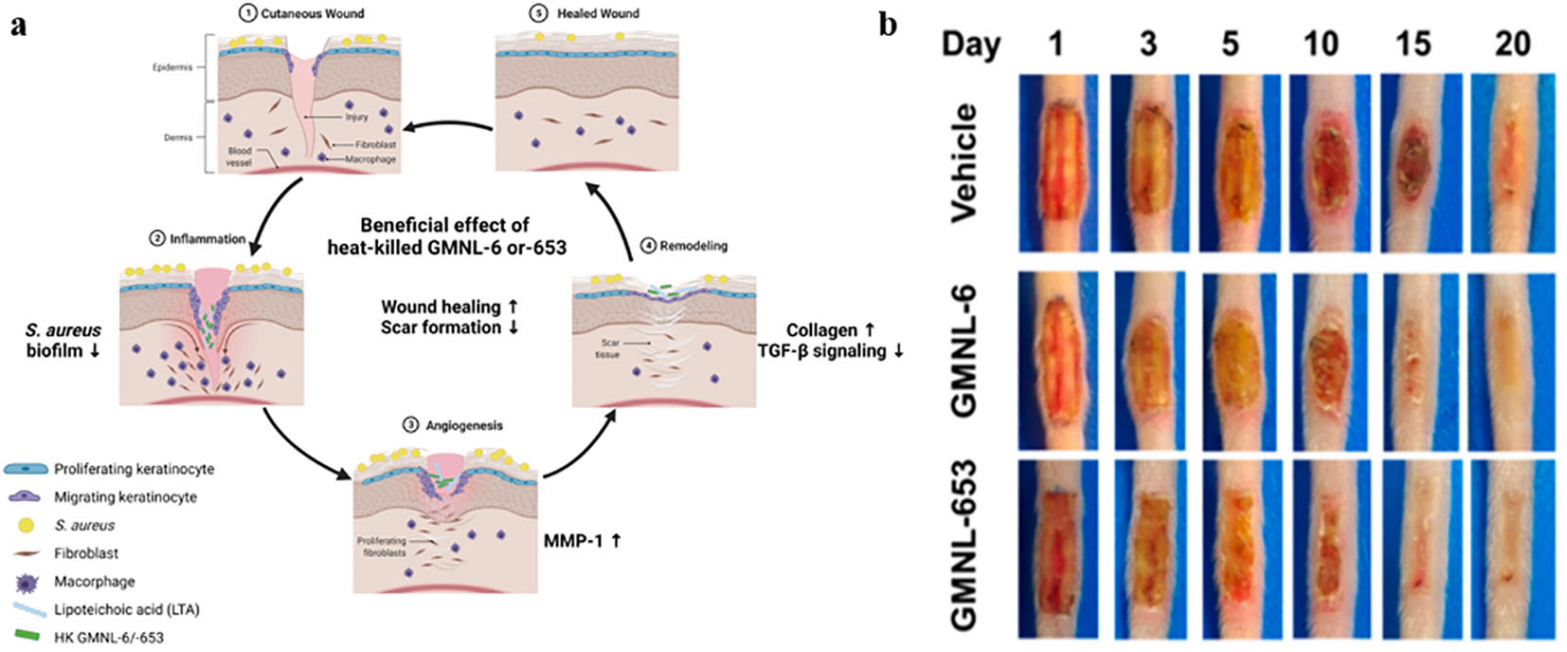
Heat-killed *Lactobacilli* probiotics preparations exhibit excellent wound healing ability in mice. **(A)** Diagram illustrating the beneficial effects of heat-inactivated GMNL-6 or GMNL-653. **(B)** The selected images of tail wounds were taken at the indicated day after wound introduction. Reprinted from Ref Tsai et al. (2021) with permission.

Microbiotherapy has significant advantages in promoting burn wound healing, mainly reflected in stimulating cell proliferation, differentiation, and tissue regeneration (Song et al., 2024). Some studies have shown that specific probiotic strains can increase collagen production, stimulate blood vessel formation, accelerate wound contraction, and promote the production of essential growth factors (**Figure 2**) (Tsai et al., 2021). Growth factors, extracellular matrix components, and other bioactive substances play a crucial role in wound healing. These molecules activate intracellular signaling pathways by binding to receptors on the cell surface (Walter et al., 2023). For example, epidermal growth factor (EGF) and TGF-β can stimulate epidermal cell proliferation and migration. Fibrinogen, collagen, and other extracellular matrix components provide a scaffold structure for cells, supporting fibroblast and epidermal cell attachment and proliferation on the wound surface, thereby promoting granulation tissue formation (Larivière et al., 2003; Zarei and Soleimaninejad, 2018; Legrand and Martino, 2022; Song et al., 2023; Fan et al., 2024). Microbiotherapy enhances these effects by regulating the expression, secretion, or activity of the aforementioned bioactive substances.

Microbiotherapy can also accelerate wound healing by improving the wound’s microcirculation, and an adequate number of new blood vessels play a key role in this process. Probiotics can significantly increase the expression levels of wound vascular endothelial growth factor (VEGF), TGF-β, angiopoietin-1 (ANG-1) and basic fibroblast growth factor (bFGF). These growth factors then bind specifically to receptors on the surface of endothelial cells in the wound, activating intracellular signaling pathways like mitogen-activated protein kinase (MAPK). This activation stimulates the proliferation and migration of endothelial cells, which gradually form tubular structures that eventually develop into mature capillaries. At the same time, the strain secretes sphingolipid metabolites, improves the local microenvironment, enhances the ability of endothelial cells to form tubes, up-regulates the expression of endothelial cell marker CD31, and further promotes neovascularization and maturation (Liu et al., 2023). Although these mechanisms show unique advantages in the preclinical experimental environment, further longitudinal studies are needed to fully translate these effects into clinical benefits for severely burned patients.

**Figure 3:**
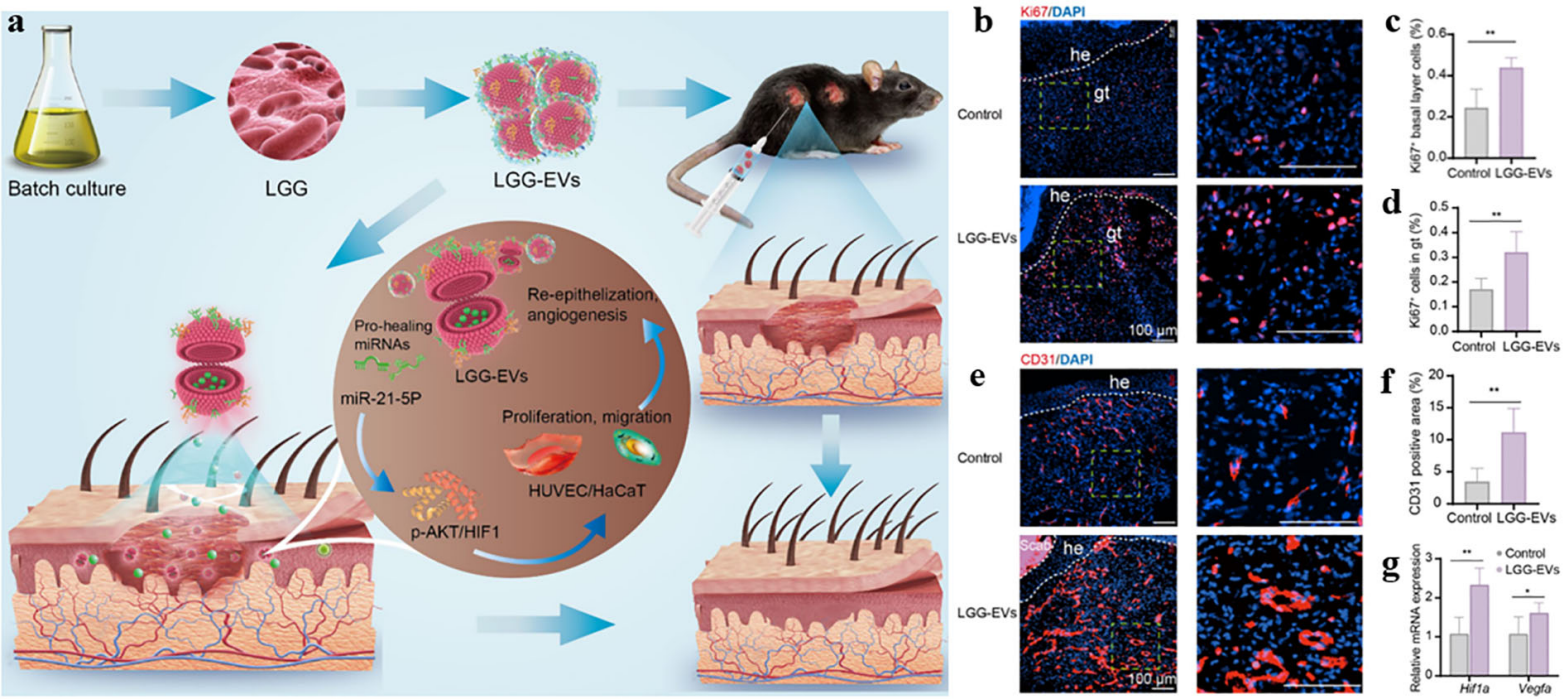
LGG-EVs application enhances wound cell proliferation and angiogenesis. **(A)** The schematic diagram. **(B)** Immunofluorescent images of Ki67 (red staining) and DAPI (blue) in the wounds on day 7 (n=5). **(C, D)** Quantification of Ki67 positive cells in the basal layer of epidermis **(C)**, and dermis **(D)**. **(E)** Immunofluorescent images of CD31 (red) and DAPI (blue) in the wounds on day 7 (n=5). **(F)** Quantification of CD31 positive area in the wounds. **(G)** RT-qPCR analysis of Hif1α and Vegfa in the wounds on day 7 (n=5). gt, granulation tissue. Reprinted from Ref Wang et al. (2024) with permission.

A concrete mechanistic study further elucidates the potential of microbial derivatives in wound repair: Wang et al. investigated the wound healing-promoting mechanism of EVs derived from *Lactobacillus rhamnosus* GG (LGG) (Wang et al., 2024). They found that these LGG-derived EVs (LGG-EVs) can deliver highly modulates miR-21-5p to endothelial cells and keratinocytes, which activates the PI3K-AKT/HIF1α signaling pathway to mediate metabolic signaling rewiring, thereby enhancing cellular proliferation and migration capacities (**Figure 3**). This study not only provides a theoretical basis for the clinical translation of probiotic-derived bacterial extracellular vesicles (BEVs) in wound repair but also expands the application scope of microbial derivatives in wound treatment. However, it should be noted that the translation from controlled animal models to the complex clinical environment of severe burns involves multifactorial challenges that necessitate more comprehensive validation.

### 2.5 Immune modulating effect

After burns, the body’s immune system enters an imbalanced state, which may manifest as immunosuppression or excessive immune activation (Burgess et al., 2022; Sierawska et al., 2022). In the early stages of acute burns, the immune system is activated to cope with trauma and infection, but if the immune response is overly intense or persists for too long, it can lead to excessive consumption of immune cells, impaired immune function, putting the body in an immunosuppressive state (Wang et al., 2022). On the other hand, excessive immune activation may also cause damage to self-tissues, further exacerbating the inflammatory response of the burn wound (Xiang et al., 2024).

Restoring immune homeostasis is crucial for burn healing, while microbiotherapy effectively helps restore the balance by regulating innate and adaptive immune responses, thereby promoting burn wound repair. Both immunoactive peptides and probiotics can regulate the proliferation, polarization and phagocytosis of macrophages, thereby contributing to skin wound repair. However, the signaling pathways involved differ substantially between these two agents. Immunoactive peptides activate TLR4 signaling to mediate macrophage proliferation and enhance phagocytic clearance of wound pathogens and necrotic debris. Concurrently, they suppress downstream NF-κB-mediated pro-inflammatory signaling, promote macrophage polarization toward the M2 phenotype, and thereby improve the local immune microenvironment (Jian et al., 2024). The regulatory mechanism of probiotics is more diverse. Some strains can regulate macrophage proliferation and phenotypic differentiation through MAPK, peroxisome proliferator-activated receptor γ (PPARγ), and other pathways. Some strains can indirectly promote wound repair by regulating intestinal flora and balancing Th1/Th2 immune response (Pradhan et al., 2019; Wang et al., 2020). Furthermore, in the early post-burn stage, pathogenic bacterial infections induce macrophages to polarize toward the M1 phenotype, which releases large amounts of pro-inflammatory factors that aggravate tissue damage and delay healing (Nishiguchi et al., 2017). Probiotic cell wall components or metabolites can inhibit the TLR4/NF-κB signaling pathway, thus preventing excessive M1 activation. Specifically, certain short-chain fatty acids in postbiotics can moderately preserve M1 function during the early healing phase, while promoting M1-to-M2 polarization through metabolic regulation in the later phase, achieving precise coordination between immune responses and tissue repair. For burn infection, bacteriophages have the effect of reshaping the immune microenvironment, by activating TGF-β1, sma- and mad-related protein 2/3 (Smad-2/3) pathway, regulating matrix metalloproteinase (MMP) balance, synergistically regulating immune homeostasis and removing drug-resistant bacteria (Aydin et al., 2025). Some phages, independent of bacteriolysis, can inhibit the transcription and expression of pro-inflammatory factors such as interleukin-1β (IL-1β), IL-6 and TNF-α, reduce neutrophil infiltration, increase macrophage recruitment, trigger specific immune signals, and inhibit inflammation amplification, thereby accurately regulating the host ‘s innate immune response (Sinha and Maurice, 2019; Suda et al., 2022). Although microbiotherapy has broad prospects as an immune regulation strategy, due to the significant individual immune heterogeneity in severely burned patients and the dynamic changes of systemic inflammatory response, how to accurately implement microbiotherapy to achieve clinical transformation still needs more rigorous clinical verification.

## 3 The main microbiotherapy for burn treatment

Probiotics are defined as live microorganisms that are beneficial to the host. Their primary use is focused on digestive health, but recent studies demonstrate that they also have potential applications for skin health and wound healing. Prebiotics, postbiotics and synbiotics have gradually garnered widespread attention in wound healing due to their unique mechanisms and biosafety (**Table 2**). In addition, common microbiotherapies for wound treatment include bacteriophages, antimicrobial peptides and other substances. These microbiotherapies have demonstrated great potential in the treatment of various traumas, such as burns, diabetic foot ulcers, and chronic wounds (Vyas and Vasconez, 2014; Rosenbaum et al., 2018; Wong et al., 2024). As a typical acute thermal injury, burn wounds are usually accompanied by large area of tissue necrosis, strong systemic inflammatory response, severe vascular injury, barrier function destruction and high risk of bacterial infection (Karna et al., 2026). The pathological mechanism of burn wounds is significantly different from that of other wounds with local ischemia, metabolic disorder or impaired repair ability. In spite of this, various wound models show some common characteristics in pathological progression, including uncontrolled microbial colonization, imbalance of inflammatory response, and instability of the repair microenvironment (Miron et al., 2023). Based on the systematic cognition of microbiotherapy accumulated in the study of various types of skin wounds, the core mechanism and repair path revealed by these studies provide key theoretical support and methodological reference for the remodeling of microecology in burn wounds.

**Table 2:** Comprehensive information comparison of probiotic-related preparations.

Category	Definition	Main ingredients	Advantages of burn scenario		Limitations of burn scenario		Literature
Probiotics	Active microorganisms, beneficial to the host, gut colonization, health regulation	*Lactobacillus*, *Bifidobacterium*, *Lactobacillus plantarum*, *Lactobacillus casei*, etc.	Intervene in gut microbiota structure, improve microbial imbalances, assist in wound infection control		Poor tolerance to the harsh wound microenvironment, limited colonization ability of the wound, strict storage condition, strain-specific dependence, unclear dosage and treatment course		(Sanders, 2008; Reid, 2016; Reid et al., 2019)
Prebiotics	Human non-absorbable food components, selective utilization by specific microorganisms, enhance gut microbiota activity	Fructooligosaccharides, galactooligosaccharides, inulin, resistant starch, polyols, etc.		High stability, resistant to high temperatures and gastric acid, easy to store and administer via enteral nutrition, no risk of live bacteria survival, broader applicability, primarily exerting effects indirectly	Cannot directly regulate the intestinal flora, dependent on the patient’s original probiotics, slow therapeutic effect		(Valcheva and Dieleman, 2016; Gibson et al., 2017; Pluta et al., 2020; You et al., 2022; Arapović et al., 2024)
Postbiotics	Non-living microorganisms or their components, beneficial to the host	Microbial cell components, peptidoglycan, active lipids, short-chain fatty acids, vitamins, organic acids, etc.		Highly safe, regulate gut microbiota balance, enhance intestinal barrier function, fast-acting, deliver anti-inflammatory and restorative effects	Clinical research limited, no clear standards, the usage thresholds are high	(Scott et al., 2022; Vinderola et al., 2022; Liang and Xing, 2023; Ma et al., 2023)	
Synbiotics	A mixture containing live microorganisms and substrates selectively utilized by the host microorganisms	Common combinations include *Bifidobacterium* + fructooligosaccharides, *Bifidobacterium* + galactooligosaccharides, and *Lactobacillus* + lactitol, etc.		Complementary synergy, enhance efficacy, address single limitations, accelerate onset, adapt to complex intestinal flora and immune requirements	Requires precise matching, high cost	(Chang et al., 2016; Gomez Quintero et al., 2022; Jangra et al., 2025)	

### 3.1 Probiotics

**Figure 4:**
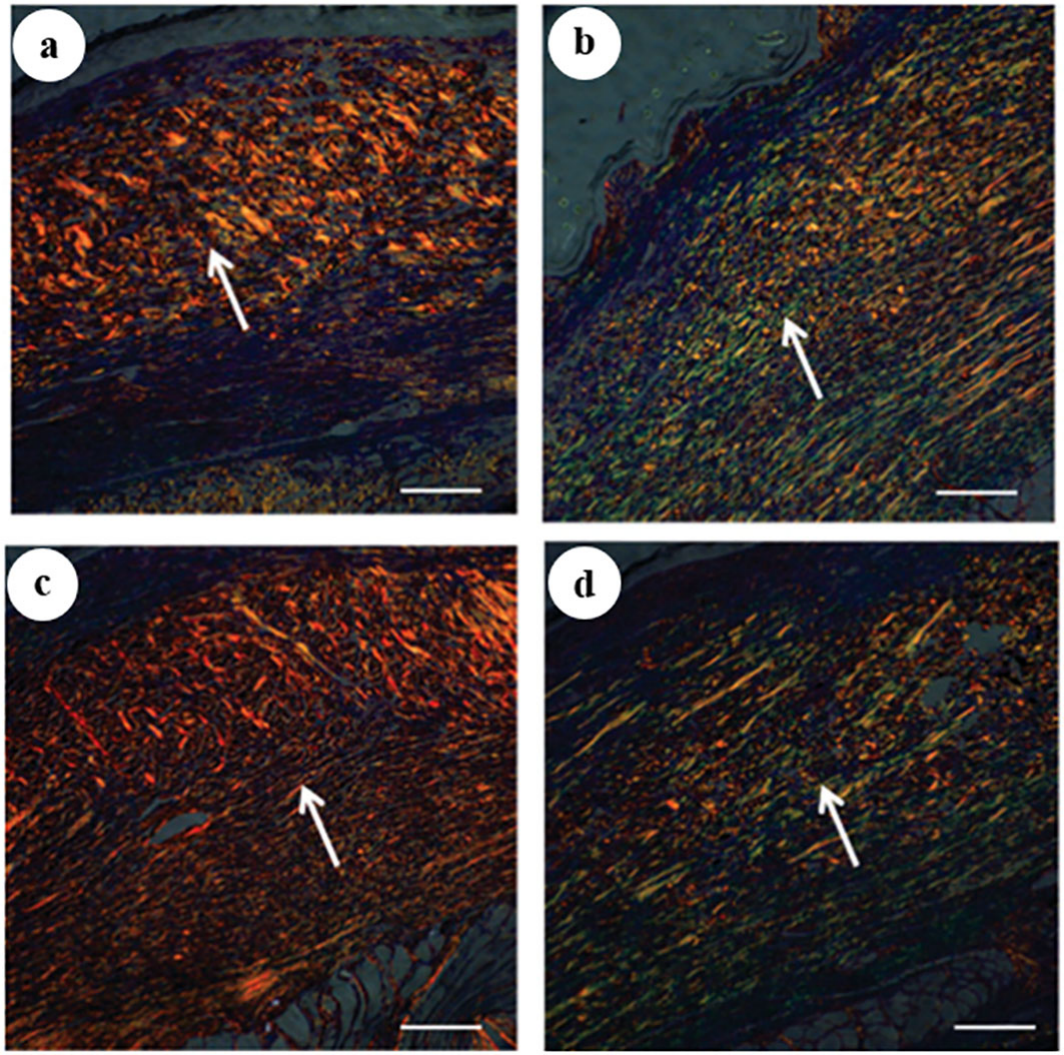
The distribution of collagen fibers stained with Sirius red under four burn wound conditions is demonstrated. **(A)** burn wound only; **(B)** burn wound + *L. plantarum*; **(C)** burn wound + *P. aeruginosa*; **(D)** burn wound + *L. plantarum* + *P. aeruginosa*. Reprinted from Ref Satish et al. (2017b) with permission.

Existing *in vivo* studies and animal experiments have revealed the possibility that probiotics can promote wound healing through multiple mechanisms (Teymouri et al., 2025). Pirouzzadeh et al. developed a novel microbial dressing carrying probiotics. The dressing employed *Lactobacillus plantarum* as its core active strain, used sodium alginate as the carrier framework, incorporated aloe vera gel and zinc chloride. Through a combination of *in vivo* and *in vitro* experiments, the authors expanded the role of probiotics from single antibacterial effects to multifunctional synergistic actions encompassing anti-infection, anti-inflammation, and promoting healing, thereby providing a complete mechanistic chain for probiotic application in burn treatment (Pirouzzadeh et al., 2025). Another study explored probiotics’ role in a key post-burn challenge: scarring. Satish et al. used a rabbit burn model to test the effect of locally applied probiotics on the severity of scars caused by *P. aeruginosa* infection in burn wounds (Satish et al., 2017b). The results indicated that *Lactobacillus plantarum* not only could effectively resist burn infection, but also could reduce scar formation of the wound (**Figure 4**). This discovery addressed the gap in earlier research, which had mostly focused on infection control but paid insufficient attention to long-term scar problems. Moreover, Barzegari et al. applied gel containing *Lactobacillus acidophilus* to a second-degree burn rat model, and found that on the 14th day of administration, inflammation at the wound site significantly decreased following *Lactobacillus acidophilus* treatment, granulation tissue formation was accelerated, re-epithelialization was significant (Barzegari et al., 2017). To systematically evaluate the existing evidence base, we summarized representative probiotic strains with documented efficacy in burn wound repair, detailing their respective research models, functional outcomes, and underlying mechanistic pathways (**Table 3**).

**Table 3:** Typical probiotics with the ability to burn wound repair.

Probiotic strain	Research model	Function	Mechanism pathway	Literature
*Lactobacillus plantarum*	Rabbit full-thickness burn + *P. aeruginosa* infection; human II/III degree burn (clinical); burn + *P. aeruginosa* aeruginosa infection in mice	Inhibiting the proliferation of wound pathogens, controlling the secondary infection after burns; reducing local inflammatory response; downregulating excessive collagen deposition and inhibiting scar tissue formation; promoting granulation tissue formation and accelerating wound healing	Secreting organic acids, competing for colonization sites, and inhibiting the growth and virulence of *P. aeruginosa*; downregulating type I collagen gene and protein expression to reduce abnormal collagen accumulation; increasing the proportion of type III collagen and optimizing collagen arrangement; enhancing phagocyte phagocytosis and inhibiting cell apoptosis	(Valdéz et al., 2005; Peral et al., 2009; Satish et al., 2017a)
*Lactobacillus acidophilus*	Bacteria were isolated from the wounds of patients with II/III degree burns; burn-derived drug-resistant *Klebsiella pneumoniae*	Broad-spectrum inhibition of common burn pathogens; inhibiting the growth of drug-resistant bacteria and destroying biofilm; regulating immunity and enhancing phagocytic activity	Secreting lactic acid, H_2_O_2_ and bacteriocins to reduce pH and directly inhibit bacteria; competing for nutrients and adhesion sites to block pathogen colonization; inhibiting biofilm formation; regulating immunity via TLR-2 and enhancing phagocytosis of macrophages and natural killer (NK) cells	(Jebur, 2010; Maryam Hayder and Baydaa A. Hassan, 2021)
*Lactobacillus casei*	Second-degree burn in rats + MDR *P. aeruginosa* infection; MDR *P. aeruginosa* from burn patients	Inhibiting the growth, adhesion and biofilm formation of MDR *P. aeruginosa* in burn wounds; reducing the local inflammatory response of burn; promoting fibroblast proliferation, granulation tissue formation and re-epithelialization, accelerating wound healing.	Secreting lactic acid, acetic acid, citric acid, succinic acid and other organic acids, reducing pH to directly inhibit bacteria; competing for adhesion sites to prevent bacteria colonization; increasing the number of fibroblasts and promoting epidermal and dermal thickening	(Abootaleb et al., 2021; Soleymani et al., 2024)
*Saccharomyces cerevisiae*	Rat back full-thickness burn model	Inhibiting burn wound infection and reducing inflammatory cell infiltration; promoting fibroblast proliferation and collagen deposition; accelerating re-epithelialization and improving wound healing rate	Acidifying the wound microenvironment and inhibiting the growth of pathogenic bacteria; promoting the expression of TGF-β1 and type I collagen, accelerating the formation of granulation tissue	(Oryan et al., 2018)
*Bacillus subtilis*	Second-degree thermal burn model of rat back; mice back hydrochloric acid chemical burn model	Inhibiting the growth, adhesion and biofilm formation of MDR bacteria; degrading eschar and removing necrotic tissue; regulating the inflammatory response; promoting collagen synthesis, angiogenesis and granulation formation; accelerating re-epithelialization	Secreting proteases and levan to inhibit bacterial growth, adhesion; activating MMPs and upregulating TGF−β1 and VEGF, promoting collagen synthesis, angiogenesis and granulation formation	(Al-Dhuayan et al., 2021; Hamada et al., 2022)
Kefir (containing *Lactobacillus*, *Lactococcus*, yeasts, etc.)	Rat burn wound model	Inhibiting *S. aureus* and *P. aeruginosa*; Reducing inflammation and promoting wound contraction; Enhancing fibroblast migration, proliferation and collagen deposition; Accelerating re-epithelialization and wound healing	Secreting organic acids, polysaccharides and bacteriocins to inhibit pathogens; Downregulating IL-1β and reducing inflammatory cell infiltration; Upregulating TGF-β1 and bFGF to promote fibroblast activity; Promoting angiogenesis and collagen synthesis	(Oryan et al., 2019)

**Figure 5:**
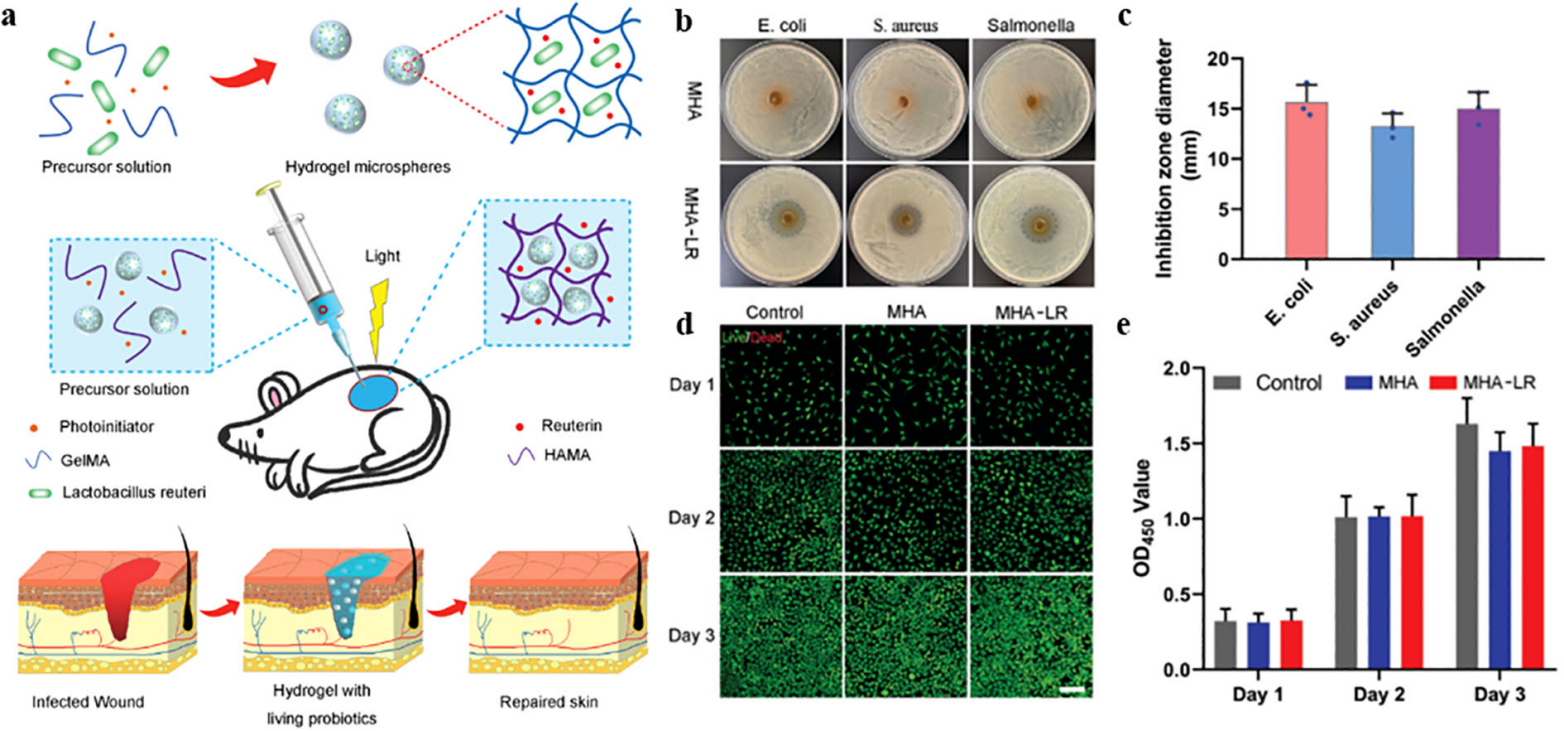
The antibacterial ability and biocompatibility of hydrogels containing *L. reuteri* encapsulated microspheres *in vitro*. **(A)** Schematic illustrations of the preparation of living probiotics hydrogels and the process of accelerating the wound healing. **(B)** Antibacterial sensitivity of hydrogels with (MHA + LR) and without (MHA) *L. reuteri* against *E. coli*, *S. aureus*, and *Salmonella*. **(C)** Inhibition zone diameters for MHA + LR. **(D)** Representative pictures of control, MHA, and MHA + LR taken by a confocal laser microscope after staining with Live/Dead Kit. **(E)** The OD value of different groups at 450 nm after incubating with CCK-8 Kit. Reprinted from Ref Ming et al. (2021) with permission.

There are also some studies that do not target burn wounds directly, but their mechanism provides important reference for burn wound healing. Xu et al. designed a high-activity probiotic hydrogel for the treatment of common acute wounds, and its matrix encapsulated *Lactobacillus paracasei* with bacterial extracellular polysaccharides (Xu et al., 2024). The study showed that bacterial extracellular polysaccharides had prebiotic properties and could promote the proliferation and metabolism of *Lactobacillus paracasei*. These probiotic hydrogels had good mechanical properties and biocompatibility, could inhibit the growth of pathogenic bacteria and maintain the stability of skin microbiota. *In vitro* and *in vivo* experiments showed that they could reduce inflammation, promote angiogenesis and collagen deposition, accelerate wound healing, and offer insights for the development of wound dressings based on live bacteria hydrogels. In addition, Ming et al. conducted a study on infectious acute wounds. *Lactobacillus reuteri* was encapsulated in hydrogel microspheres through emulsion polymerization, and then formed a hydrogel dressing through the covalent crosslinking of methylacrylate-modified hyaluronic acid (Ming et al., 2021). Through *in vitro* and *in vivo* experiments and animal experiments, it was proven that this dressing could effectively kill harmful bacteria (**Figure 5**) and reduce inflammatory cell infiltration. This study also opened up new avenues for the application of live bacteria in the treatment of infected wounds and tissue engineering. However, despite these successful animal studies, a significant challenge remains in ensuring the viability of these live bacteria when exposed to the cytotoxic environment of severe clinical burn exudates.

### 3.2 Prebiotics, postbiotics, synbiotics

The Global Prebiotic Association defined prebiotics in 2024 as compounds or ingredients used by microbial communities to offer health and performance benefits. Their main function is to regulate the makeup and activity of microbial communities in specific spots causing physiological effects (Deehan et al., 2024). Severe burns can easily induce intestinal flora imbalance and cause damage to the intestinal mucosal barrier. As nutritional substrates for probiotics, prebiotics exert their effects by promoting the proliferation of intestinal native probiotics and stimulating metabolite production. Through these actions, they indirectly enhance intestinal barrier function, reduce endotoxin absorption, and suppress excessive inflammation, thereby establishing a more stable microenvironment to facilitate burn wound healing (Baquerizo Nole et al., 2014). Prebiotics could also affect skin health through oral intake and local topical application. Research showed that topically applied prebiotics can bidirectionally regulate skin microbiota by selectively nurturing beneficial bacteria and inhibiting pathogenic bacteria (Afzal et al., 2025). Meanwhile, they can reduce pathogenic bacterial colonization and virulence by interfering with biofilm formation or modulating the local microenvironment (Wei et al., 2023). Prebiotics can also regulate NF-κB signaling pathway in HaCaT keratinocytes, promote cell migration and differentiation, and have good biocompatibility: They can adsorb wound exudate, providing a suitable environment for fibroblast proliferation (Zeng et al., 2025). In conclusion, prebiotics possess characteristics such as regulating the balance of gut microbiota, protecting the mucosal barrier, and promoting cell repair. These characteristics also enable prebiotics to exhibit unique advantages in the burn treatment field distinct from others, providing important directions for the development and optimization of subsequent related therapeutic schemes.

Postbiotics refer to biologically active substances such as cell wall components, metabolic products, or cellular lysates from probiotics after they undergo fermentation, metabolism, or death. Compared to probiotics, they do not need to survive to take effect, have strong stability, and are more suitable for the complex microenvironment of burn wounds (Ekrami et al., 2024). Currently, numerous studies have demonstrated that postbiotics exhibit good microbial activity in the process of repairing various skin wounds, including immune regulation and biofilm interference (Chang et al., 2025; Mohammadhosseinzadeh et al., 2025; Zhang et al., 2025b). These research outcomes not only broaden the application scope of postbiotics in biological therapeutics but also indirectly indicate their potential values in treating more complex burn wounds. Studies have proven that postbiotics can interfere with the biofilm formation of *P. aeruginosa*, reducing its adhesion ability, thereby reducing the risk of infection in wounds (Azami et al., 2022). In addition, Ishi et al. used a mouse full-thickness skin wound model systematically to demonstrate that heat−killed *Lactobacillus plantarum* accelerated wound healing by regulating macrophage polarization and relying on the CARD9-mediated signaling pathway (Ishi et al., 2023). This provides reliable support for the use of postbiotics in wound treatment. Golkar et al. clarified the core pathway of postbiotics in wound treatment through the “antibacterial - anti-inflammatory - pro-regenerative” triple mechanism by conducting *in vivo* experiments and acute non-infected wound model experiments in rats. Especially, they confirmed that postbiotics derived from *Lactobacillus reuteri* and *Bacillus subtilis* sp. *natto* showed superior therapeutic efficacy, thus offering a new intervention strategy with high safety for wound treatment (Golkar et al., 2021). Postbiotics rely on their unique biological activity and good stability, showing great application potential in the treatment of burns and other complex wounds. With further in-depth research into their mechanism of action and continuous advancement in clinical translation, postbiotics are expected to become a new type of safe, effective, and promising microbiotherapy approach in the future field of wound treatment.

Synbiotics, as a combination of probiotics and prebiotics, also show unique potential in burn treatment. Studies show that the optimization of gut microbiota is closely related to the reduction of systemic inflammatory levels (Bindels et al., 2016; Shimizu et al., 2021; Li et al., 2023). By providing probiotics and their necessary nutrients for growth, the synbiotic can function in regulating gut microbiota and enhancing immune function, thereby indirectly promoting the healing of burn wounds (Shimizu et al., 2013; Khursheed et al., 2022; Lee et al., 2024). In the field of burn wound treatment, synbiotics utilize the mechanism of enhancing probiotic functions and boosting prebiotic synergy, serving as an important potential solution for combating MDR bacteria and promoting burn wound healing. A study used commercial synbiotics containing different probiotic strains. By collecting the cell-free supernatant (CFS) that has soluble factors secreted by the probiotics, researchers found that this CFS can directly inhibit the growth of MDR *P. aeruginosa* by secreting metabolites such as organic acids and bacteriocins, and can also block the quorum sensing system of pathogenic bacteria, thereby reducing their biofilm formation and toxin secretion (Soleymani et al., 2024). This research provides a basis for using synbiotics as components in antibacterial dressings for burn wounds and as an auxiliary treatment for burn wound infections. In a recent study, a composite freeze-dried preparation with prebiotics and probiotics showed wound-healing effects similar to those of the widely used sulfadiazine silver ointment. The results suggest that prebiotics can significantly boost the activity of probiotics and work together to regulate burn wound microecology and the inflammatory response. In animal tests, this preparation was found to greatly increase the closure rate of infected burn wounds. Histological analysis also confirmed it could promote epithelial regeneration and cut down on inflammatory cell invasion (Hassaninejad Farahani et al., 2023). This research systematically reveals the multi-faceted mechanisms of synbiotic therapy for infectious burn wounds, and also provides a solid foundation for the future development and mechanistic exploration of similar synbiotic formulations.

### 3.3 Bacteriophages

**Figure 6:**
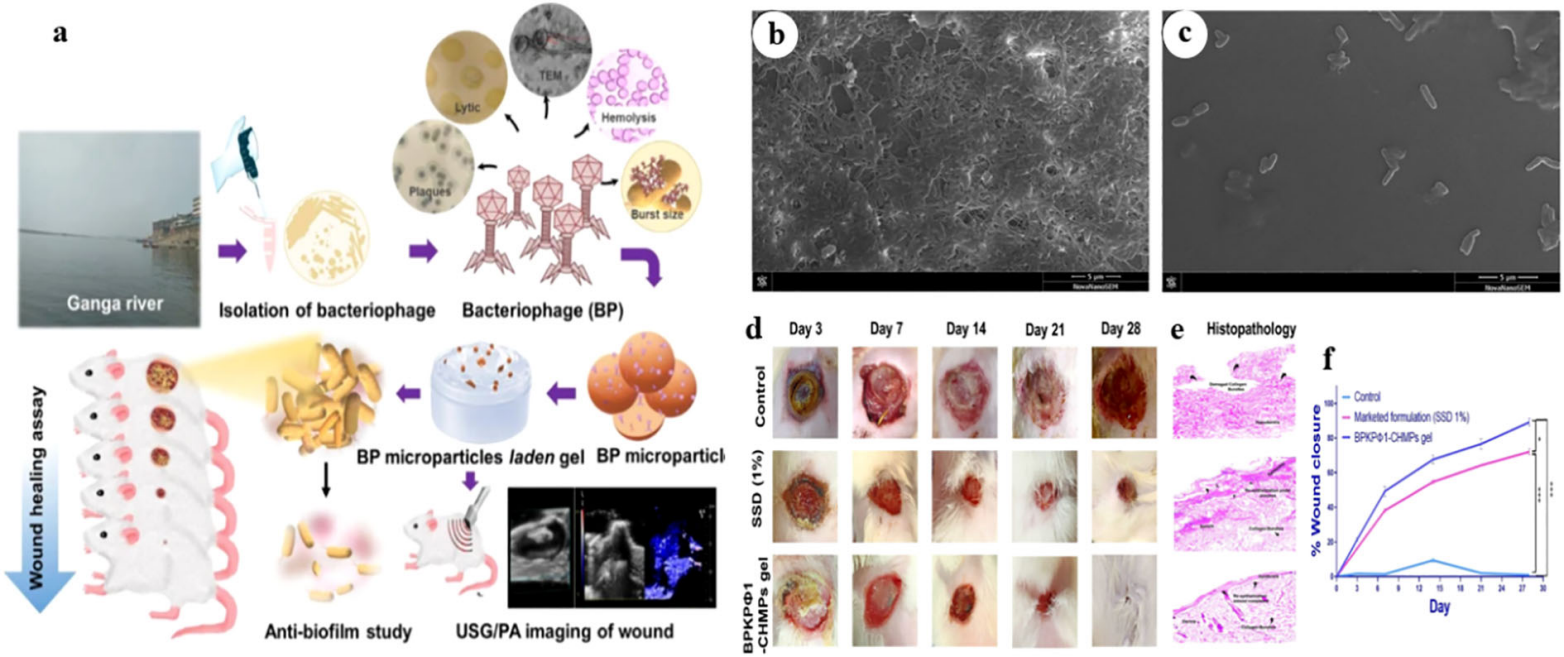
**(A)** The schematic diagram. **(B)** Biofilm before bacteriophage treatment and **(C)** after bacteriophage treatment. **(D)** Wound status on different days 3, 7, 14, 21, and 28. **(E)** Histopathology examination of the skin. **(F)** Graphical representation of % wound closure. Reprinted from Ref Dehari et al. (2023a) with permission.

**Figure 7:**
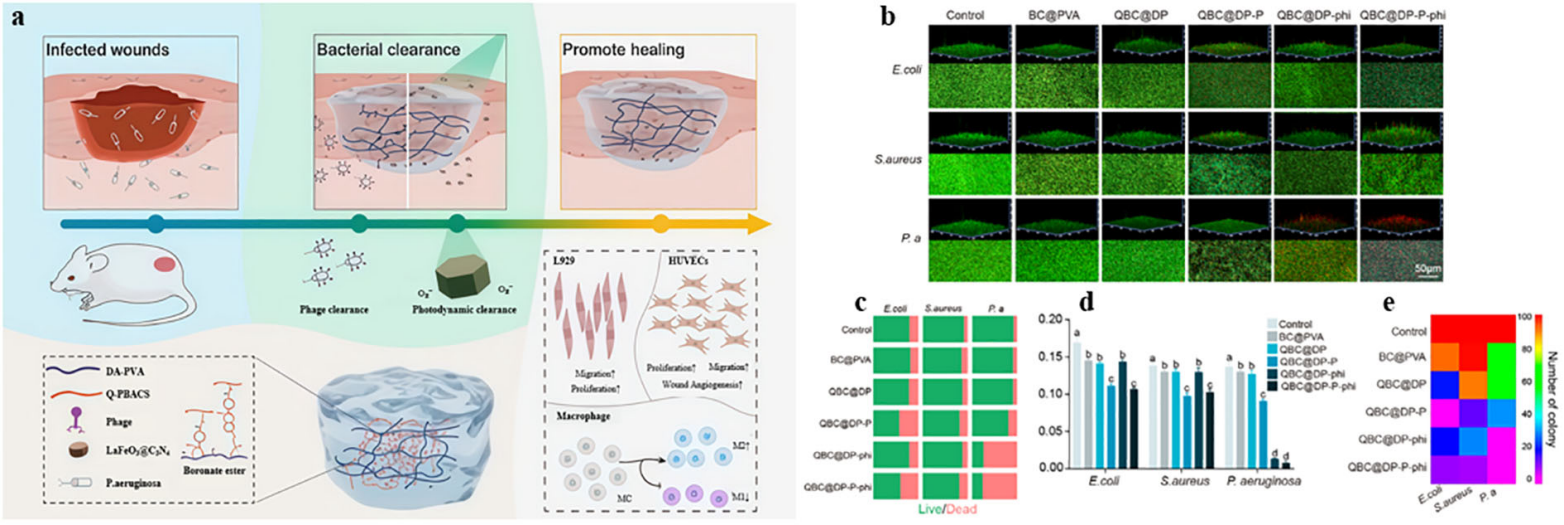
**(A)** The schematic diagram. **(B)** Bacterial live/dead staining. **(C)** Bacterial survival (semi-quantitative). **(D)** Bacterial inhibition effect. **(E)** Agar plate count. Reprinted from Ref Quan et al. (2025)with permission.

Bacteriophages are viruses that specifically infect bacteria, killing them by attaching, injecting genetic material, and lysing bacteria (Lv et al., 2023). Due to their unique mechanism, they function as critical tools for tackling drug-resistant bacteria and aiding wound healing. Although bacteriophages do not directly take part in tissue regeneration, they facilitate infection control and reduce inflammation, making it easier for skin cells to proliferate and migrate. In burn wound treatment, bacteriophage therapy has shown promising preclinical efficacy against MDR bacteria like *P. aeruginosa* and *S. aureus* (Ata Vural et al., 2025). This is because its mechanism of action does not depend on inhibiting bacterial metabolic processes; instead, it works by directly attacking bacterial cells (**Figure 6**), so it can effectively handle drug resistance issues (Dehari et al., 2023a). A study showed that bacteriophages exhibited significant lysis activity against resistant strains. Using plaque assays, the study found that bacteriophages could significantly reduce bacterial concentrations within several hours, demonstrating their potential role in the acute phase of burn infections (Li et al., 2022a). Furthermore, Quan et al. constructed a peptide-enhanced bioactive hydrogel combined with a photodynamic-phage synergistic antibacterial therapy system (QBC@DP-P-phi) (Quan et al., 2025). This system uses the lytic *Pseudomonas aeruginosa* phage phipa10 (phi10) as a specific antibacterial component, which can precisely eliminate *P. aeruginosa* at the wound site to rapidly reduce the local bacterial load, and ultimately achieve efficient healing of infected wounds (**Figure 7**), providing a new strategy for wound treatment. Burn wound surfaces are often colonized by biofilm, thereby increasing treatment difficulty. One *in vitro* study showed that bacteriophages could significantly reduce bacterial survival rates by degrading the extracellular polysaccharide matrix of biofilms. Researchers found that bacterial counts decreased by about 2–3 log units after bacteriophage treatment, which indicated that bacteriophages had potential in clearing deep burn wound infections (Thung et al., 2019). Additionally, in a clinical trial, 27 burn patients received bacteriophage therapy for MDR *P. aeruginosa* infection. The research team isolated bacteriophages targeting this strain from environmental samples and prepared a “bacteriophage cocktail”, which is a combination of multiple bacteriophages, and administered it via local application and intravenous injection (Jault et al., 2019). The results showed that the bacterial load at the infection site of the patients significantly decreased, wound healing progressed faster, and importantly, no obvious side effects occurred. Bacteriophage therapy for burn wounds showed efficient lysis and biofilm clearance in *in vitro* studies. Clinical trials further confirmed its safety and initial efficacy (Morozova et al., 2018; Duplessis and Biswas, 2020). Although current applications are limited, its unique advantage against resistant bacteria makes it a potential breakthrough point for burn infection treatment (Walter et al., 2024). However, the transition from these successful preliminary trials to routine clinical management is hindered by several critical bottlenecks. First, the high degree of “phage-host specificity” acts as a double-edged sword; while it protects commensal flora, it necessitates rapid, real-time diagnostic tools to match specific phages to a patient’s unique bacterial isolate—a core requirement for personalized burn therapy. To overcome these obstacles, future research should prioritize the development of “broad-spectrum phage cocktails” and the exploration of “genetically engineered phages” with reduced immunogenicity. Furthermore, the field must establish standardized pharmacological protocols that define precise dosing regimens and optimal delivery intervals within existing standards of care, such as integration with surgical debridement, to ensure that bacteriophage therapy plays a transformative role in future clinical practice (Sandhu and Parida, 2026). With further research and technological improvement, bacteriophage therapy is expected to play a greater role in clinical practice.

### 3.4 Other microbiotherapies

**Figure 8:**
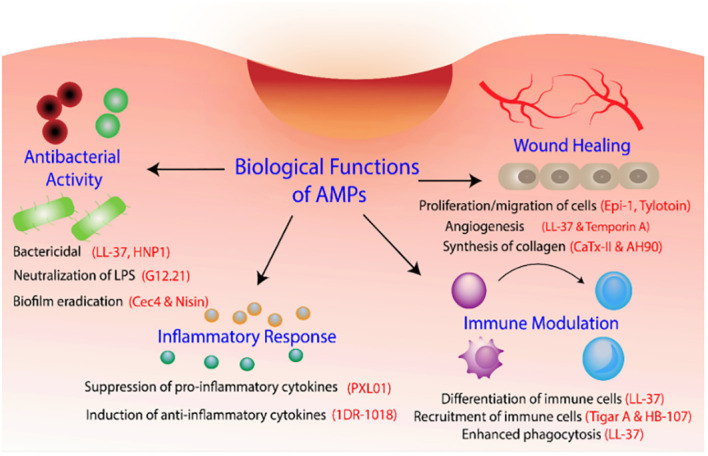
Multifunctional role of antimicrobial peptides in restoring skin homeostasis. Including limited to clearance of bacteria, modulation of inflammation, and immune response while promoting angiogenesis and tissue regeneration. Reprinted from Ref Haidari et al. (2023) with permission.

Other microbiotherapies also have application value and broad prospects in the field of burn wound treatment. Antimicrobial peptides are short-chain peptides produced by microorganisms or host cells. Due to their broad antimicrobial spectrum, low toxicity, and rapid bactericidal ability (**Figure 8**), antimicrobial peptides have the potential to become the ideal choice for preventing and treating burn infections (Bhat and Milner, 2007; Haidari et al., 2023). For example, lipopeptides produced by *Bacillus subtilis* are effective against both Gram-positive and Gram-negative bacteria (Wu et al., 2019). Antimicrobial peptides are ideal for treating skin infections and wounds. Not only can they quickly kill pathogens by disrupting the integrity of bacterial cell membranes, but they can also induce cell migration and proliferation, angiogenesis, and control immune responses (Cai et al., 2025; Zhang et al., 2025a). A study made antimicrobial peptides from microbial sources into a nanogel coating. The study found it could significantly reduce the rate of wound infection and speed up healing (Li et al., 2022c). Other studies also found that local application of antimicrobial peptides avoided the side effects possibly brought by systemic administration (Qin et al., 2025; Yang et al., 2025c). This made them particularly suitable for the acute stage of burns. In addition to antimicrobial peptides, numerous other microbiotherapies provide novel pathways to achieve more efficient and safer wound healing. As multidisciplinary integration advances, these microbiotherapies are expected to move beyond the laboratory phase. While antimicrobial peptides and other derivatives provide novel pathways for treatment, it must be recognized that their therapeutic potential in human burn treatment is still largely in the preclinical stage. Future research must address issues such as high production costs, possible proteolytic degradation in the wound bed, and the need for large-scale clinical trials to truly advance to routine clinical applications.

## 4 The combined application and synergistic effect of microbiotherapy

Burns are an extremely challenging and complex issue in the medical field, with their treatment difficulty rising exponentially as burn depth and total surface area increase (Liu et al., 2025). When treating severe burns and accompanying infection risks, the complexity of treatment is particularly prominent. Treatment must not only balance wound debridement and infection control but also promote tissue regeneration simultaneously. Traditional treatments like systemic antibiotics and local application of silver sulfadiazine often face challenges such as resistance, tissue toxicity, and delayed healing (Li et al., 2025a, b). Given this context, microbiotherapies represented by probiotics and bacteriophages have gradually gained widespread attention in their application to burn treatment. However, single microbiotherapies have limitations, including being easily influenced by specific environmental conditions and having incomplete treatment coverage. These limitations prompt researchers to explore the combined use of microbial agents, thereby better achieving synergistic anti-infection effects. Furthermore, combining microbiotherapies with traditional treatments shows significant potential for synergistic effects.

The combined application of probiotics and bacteriophages is one of the highly promising microbiotherapy strategies. Probiotics regulate skin microbiota to create a favorable environment for wounds, while bacteriophages directly clear pathogenic bacteria. This synergistic effect accelerates wound healing, reduces infection risk, and partially replaces antibiotic use, thereby offering new solutions to drug resistance (Forsyth et al., 2023; Guan et al., 2024). In addition to the combination of probiotics and bacteriophages, the role of postbiotics in enhancing the efficacy of antibiotics has also been scientifically proven. A study indicated that postbiotics could enhance antibiotic efficacy by modulating membrane permeability, disrupting biofilms, or altering bacterial communication systems. Low cytotoxicity and pathogen-specific responses in experiments demonstrate that postbiotics are not only safe but also allow for tailored targeting based on therapeutic needs. Particularly when combined with antibiotics like linezolid or amikacin, postbiotics form low-toxicity, synergistic treatment strategies, further expanding options for burn infection management (Yaprak Çolak and Duran, 2025). Another study investigated the therapeutic efficacy of combining bacteriophages with antibiotics against MDR *P. aeruginosa*. Three distinct bacteriophages were isolated and screened from environmental sources. *In vitro* comparisons revealed that the dual bacteriophage-antibiotic combination demonstrated the highest bactericidal efficacy. Although this synergistic strategy has significant potential in the treatment of drug-resistant bacterial infections, its clinical transformation process still depends on rigorous *in vivo* experimental verification. In addition, overcoming the technical bottlenecks associated with phage stability is a key prerequisite for ensuring the feasibility of this therapy in future clinical practice (Aghaee et al., 2021). Notably, integrating microbiotherapies with conventional burn treatments maximizes the advantages of both approaches. This approach preserves the role of traditional methods in basic care or acute interventions while leveraging microbiotherapies to enhance treatment precision and reduce side effects, ultimately forming a comprehensive and highly effective integrated treatment plan. For instance, during an acute infection stage, antibiotics and bacteriophages can be used to rapidly eradicate bacteria, while probiotics and synbiotics may then be applied to promote wound healing and immune recovery (Abedon, 2019). In terms of clinical translatability, bridging microbial ecology with traditional surgical practice represents a pivotal shift toward precision burn care, offering a sophisticated framework for personalized wound management and accelerated functional recovery. As the field moves toward personalized therapy, optimizing these combinations based on the patient’s specific wound microenvironment will be essential. Future research must now focus on the logistical aspects of these combined therapies, including the formulation of stable co-delivery systems, the assessment of cost-effectiveness compared to standard care, and the establishment of regulatory frameworks for “living” or “hybrid” drug products. Such advancements hold the promise of revolutionizing burn care, propelling the field into a new era of enhanced efficiency and clinical precision.

## 5 Current challenges and future prospects

The stability and safety issues of agents in burn wound microbiotherapy are core bottlenecks restricting their application, and establishing a targeted biosafety assessment system has become an urgent priority. In terms of agent stability, factors in the complex burn wound microenvironment including high temperature, pH changes, protease activity, and wound exudate may all inactivate microbiotherapeutic agents, which is one of the key issues facing this therapy. In terms of safety, microbiotherapy has multiple limitations: the use of exogenous microbial agents may trigger host immune reactions. For example, bacteriophages may induce antibody production thus reducing efficacy (Lin et al., 2022; Guo et al., 2024). Some agents may exhibit toxicity to human cells under high doses, especially for large burn wounds with severe infection, excessive doses may lead to cell damage (Rima et al., 2021; Wang et al., 2021; Bucataru and Ciobanasu, 2024). Beyond these general toxicity risks, the application of microbiotherapy faces unique safety challenges. For severely burned patients, the integrity of the skin barrier has been destroyed, and the risk of exogenous live probiotic strains or other active microorganisms entering the blood circulation through the wound has increased significantly, which can easily lead to infections such as bacteremia or sepsis (Hosoda et al., 2025). In addition, the colonization of live bacteria in the wound is difficult to be accurately controlled, and its excessive proliferation may destroy the microecological balance of the wound. Due to the lack of uniform dose and frequency standards, the potential pathogenicity and long-term safety of live bacteria after long-term colonization *in vivo* are still uncertain, which further increases the complexity of clinical risk management and control (Wang et al., 2025; Zhou and Zhou, 2025). Therefore, the safety evaluation system for *in vivo* microbiotherapy should not be limited to traditional toxicity tests, and a multi-dimensional monitoring framework covering systematic monitoring, immunogenicity evaluation and long-term colonization tracking must be constructed. Currently, safety evaluations for microbial agents used in burn care lack a standardized framework, unlike vaccines which undergo rigorous Phase I-III trials and long-term follow-up. Given the unique nature of burn treatment, biosafety assessments must establish corresponding protocols (Locker et al., 2024). Specifically, short-term toxicity testing should incorporate dose-escalation studies tailored to the characteristics of burn wounds. Immunological safety screening must prioritize evaluating the immunological interactions between the formulation and the host. Long-term monitoring requires tracking subjects for 6 to 12 months to comprehensively assess long-term effects.

Effectively delivering microbial agents to burn infection sites is a crucial challenge lacking mature solutions, requiring systematic dosage form screening and multi-stage validation, with several delivery-related problems awaiting further research. Factors such as wound exudate, local environment of bacterial infection, and blood supply at the wound site will all affect delivery and absorption of microbial agents. Although intelligent drug delivery systems such as nanoparticles and microcapsules show good effects in the laboratory, applying these technologies to actual clinical treatment of burn wounds still faces many technical and practical challenges (Feng et al., 2025; Gullifa et al., 2025; Mavaddat and Zandkarimi, 2025). This highlights the need for a systematic screening approach to identify the optimal dosage form. Such a screening process is critical to addressing delivery inefficiencies, as mismatched formulations often worsen agent inactivation or poor tissue penetration. Initial *in vitro* simulation tests should evaluate the stability, sustained release capacity, and antibacterial activity of different formulations under simulated burn wound conditions. Subsequently, the best-performing dosage form should be tested in burn-induced animal models to validate its ability to penetrate the biofilm and its tissue biocompatibility. Finally, clinical studies should validate its usability and preliminary efficacy in burn patient populations to ensure suitability for real-world clinical settings. Key issues requiring further investigation include: preventing formulation inactivation during the delivery process, ensuring sustained effective drug concentrations at the wound site, and minimizing collateral tissue damage (Whittam et al., 2016).

Compared with traditional drugs, the production process of microbial agents is more complex, and quality control standards are stricter. Determining doses and defining application scopes require more precise research, all of which lead to slow translation from laboratory research to clinical practice. The dose determination of microbial agents is not as simple as that of traditional drugs: traditional drug doses are usually set based on pharmacokinetic and pharmacodynamic characteristics, while microbial agents—due to their high bioactivity and variability—often require individualized adjustment (Lathia and Watson, 2024). For example, bacteriophage doses and regimens require adjustment according to the type of infected bacteria, infection site, and patient’s immune status. This complexity poses additional challenges in clinical application and slows the process of large-scale adoption. Although *in vitro* experiments and animal models have demonstrated microbiotherapy’s potential, large-scale clinical trials for human burn infections remain scarce—most studies enroll fewer than 100 patients and lack long-term efficacy and safety data (Yu et al., 2023). This gap not only hinders widespread clinical acceptance but also delays regulatory approval and clinical translation. Future efforts must therefore prioritize large-scale, long-term follow-up clinical studies, while accelerating the development of dedicated approval standards and evaluation systems for microbial agents by regulatory agencies to bridge the “laboratory-clinic” divide (Aziz and Zaidi, 2025; Fuerst-Wilmes et al., 2025). Additionally, future research needs to deepen understanding of microbiotherapy’s specific mechanisms and optimize its production and quality control standards.

A key misconception that requires clarification is that in burn treatment, microbiotherapies serve as complementary approaches to antibiotics, not replacements. In severe burns, antibiotics remain indispensable for rapidly controlling life-threatening systemic infections, while microbiotherapies excel at addressing the limitations of antibiotics (Ramkissoon et al., 2026). Establishing this complementary relationship is a crucial prerequisite for avoiding overreliance on a single therapy and maximizing treatment efficacy.

Microbiotherapy demonstrates great potential in burn treatment, but it still encounters numerous challenges in practical clinical applications. These challenges span formulation stability, safety, delivery, and dosage, demanding in-depth research and technological innovation (Kelly et al., 2020). Additionally, the standardization of microbiotherapy, regulatory approval procedures, and the advancement of large-scale clinical trials urgently need strengthening (Rodriguez et al., 2025). Only through multidisciplinary collaboration, technological breakthroughs, and policy support can we gradually overcome existing obstacles, allowing microbiotherapy to become a safe, effective, and scalable tool in burn treatment. In the future, as research deepens and technology progresses continuously, microbiotherapy is expected to play a more significant role in burn infection management, providing patients with better treatment options and improved rehabilitation outcomes.

## 6 Conclusion

This review systematically summarizes recent advances in microbiotherapies for burn wound treatment, focusing on three primary application forms: probiotics, bacteriophages, and microbial metabolic derivatives. These therapeutic approaches, whether used alone or in combination, have demonstrated significant potential to synergistically repair burn wounds through antibacterial effects, modulation of inflammatory responses, promotion of wound healing, and restoration of immune homeostasis, among other mechanisms. Existing *in vivo* research findings further validate the feasibility of microbiotherapies, marking a gradual transition from laboratory studies to preclinical models early and clinical applications. Despite existing challenges, microbiotherapy provides a novel, effective approach for burn wound management, expanding treatment options and offering new solutions to antibiotic resistance in burn infections.
